# Optimization and Modelling of the Physical and Mechanical Properties of Grass Fiber Reinforced with Slag-Based Composites Using Response Surface Methodology

**DOI:** 10.3390/ma17153703

**Published:** 2024-07-26

**Authors:** Jiale Ma, Long He, Zhixin Wu, Jiarui Hou

**Affiliations:** Green Building Autonomous Region Key Laboratory of Higher Education, School of Architecture, Inner Mongolia University of Technology, Hohhot 010051, China; 20221100460@imut.edu.cn (J.M.); 20221800651@imut.edu.cn (Z.W.); 20231100462@imut.edu.cn (J.H.)

**Keywords:** grass fiber, mechanical properties, physical properties, slag-based, response surface methodology, optimization

## Abstract

The construction industry’s high energy consumption and carbon emissions negatively impact the ecological environment; large-scale construction projects consume much energy and emit a significant amount of CO_2_ into the atmosphere. Statistics show that 30% of energy loss and 40% of solid waste in the construction industry are generated during construction. Therefore, reducing emissions during construction has significant research potential and value. Many scholars have recently studied eco-friendly building materials to facilitate the use of high-carbon emission materials like cement. Adding fibers to composite materials has become a research hotspot among these studies. Although adding fibers to composite materials has many advantages, it mainly reduces the compressive strength of the composite material. This research used the response surface methodology (RSM) to optimize the raw material ratios and thus improve the performance of plant fiber composite materials. Single-factor experiments were conducted to analyze the effects of grass size, grass content, and quicklime content on the composite materials’ compressive strength, flexural strength, and water absorption. The influencing factors and levels for the response surface experiment were determined based on the results of the single-factor analysis. Using the response surface methodology (RSM), a second-order polynomial regression model was established to analyze the interaction effects of the three factors on the composite materials’ compressive strength, flexural strength, and water absorption rate. The optimal ratio was determined: the optimized options for grass size, grass content, and quicklime content are 2.0 mm, 8.2 g, and 38 g, respectively. The actual values of compressive strength, flexural strength, and water absorption rate of the composite materials made according to the predicted ratio are 11.425 MPa, 2.145 MPa, and 21.89%, respectively, with a relative error of 8% between the actual and predicted values. X-ray diffraction and scanning electron microscopy were also used to reveal the factors contributing to the relatively high strength of the optimized samples.

## 1. Introduction

The emission of greenhouse gases is one of the most severe environmental problems globally, as it is the primary cause of global warming. Carbon dioxide accounts for 82% of total greenhouse gas emissions [1], with the cement industry producing 7% of the world’s anthropogenic carbon dioxide despite emitting almost no other greenhouse gases [2]. The production of Portland cement significantly contributes to global warming. Consequently, finding alternatives to cement has become a significant concern in the construction industry. Since the 1960s, extensive research has focused on developing cement-free slag or geopolymer-based alkali-activated composites. These studies have examined these materials’ mechanical and chemical properties to enhance performance and reduce cement use in the construction industry [3,4,5,6,7,8].

Alkali-activated slag can be better applied to building materials. Alkali-activated slag materials exhibit mechanical properties similar to cement-based materials [3,5]. The primary advantage of alkali-activated slag is its environmental friendliness, as alkali-activated slag composites produce less carbon dioxide compared to cement-based composites. Additionally, recent studies have reported that alkali-activated slag-based cementless fiber-reinforced composites can achieve high tensile strain capacity and tensile strength [9,10,11], laying the foundation for replacing cement-based composites with slag-based composites. However, alkali-activated composites exhibit brittleness and are prone to cracking [12,13]. Nonetheless, incorporating appropriate fibers can improve the flexibility of alkali-activated cementless composites.

Previous studies have added synthetic fibers such as tire shreds, PVA fibers, basalt fibers, glass fibers, and polypropylene fibers to composites. Extensive research has been conducted on tire shred-reinforced composites [14,15]. Tyre shred-reinforced composites have been proven to improve the matrix material’s flexibility, toughness, thermal conductivity, and acoustic properties, but their compressive and flexural strengths are compromised [16]. Lee et al. explored the feasibility of developing strain-hardening fiber-reinforced cementless composites using mortar based on alkali-activated ground granulated blast furnace slag (GGBS) and PVA fibers [10]. The results showed a tensile strain-hardening behavior, and up to 4.5% flexibility could be achieved [12]. Similar results were obtained in studies using basalt fibers, where fiber-reinforced composites exhibited better fracture performance than conventional Portland cement, and the addition of fibers also reduced the presence of cracks. Incorporating these synthetic fibers into composites can improve their mechanical, physical, and processing properties. However, traditional synthetic fibers have poor biodegradability and cause severe environmental pollution, leading to widespread interest in research on new composite reinforcements based on biodegradable resources [17]. In addition to being lightweight, cost-effective, abundant, and biodegradable, natural plant fibers possess high specific strength and modulus, making their composites a research focus for scholars in recent years [18].

In existing studies, researchers have used many materials such as wood [19,20], sisal [21,22,23,24], hemp [25,26], coconut [22,27,28,29], bamboo [30], wheat straw [31], and rice straw [32] in composites. Zhao et al. [19] studied rubberwood particle-reinforced composites. They found that adding rubberwood particles improved the composites’ flexural toughness and impact resistance, reduced weight, and enhanced thermal insulation and energy savings. Silva et al. [24] studied the physical and mechanical properties of durable sisal fibers added to composites through direct tensile and bending tests to determine their mechanical properties under static loads. They assessed the physical properties of the composites through drying shrinkage rate, capillary water absorption, and water tightness. The studies showed that sisal composites performed better in tension and had twice the toughness of ordinary composites; physical performance tests showed that sisal composites had reasonable drying shrinkage rates and water tightness. Hemp fibers have excellent mechanical properties, and their composites possess good mechanical performance on a green and environmentally friendly basis, with some properties almost comparable to those of synthetic fiber composites [17]. Millogo et al. [33] studied the addition of hemp fibers to composites. They found that adding 0.2–0.6 wt.% of 30 mm fibers could reduce the pore size in PAB (Pressed Adobe Blocks) and improve its mechanical properties. However, adding 0.8 wt.% of 60 mm fibers negatively impacted compressive strength.

Sedan et al. [26] found that hemp fiber-reinforced composites significantly improved flexural strength in their mechanical properties. Lee et al. [34] studied the compressive and flexural properties of hemp fiber-reinforced composites and found that the flexural toughness of hemp fiber-reinforced composites increased by 144%. Coconut fibers showed better flexural strength than synthetic fibers [35]. Wang et al. [29] reported that the flexural toughness of coconut shell fiber composites increased more than tenfold. In another study, Chin et al. [36] found that bamboo fibers have a negative effect on compressive strength and split tensile strength, but the negative effect of bamboo fibers decreases with the increasing age of concrete. Petrell et al. [31] investigated the production of sustainable cement mortars from wheat straw fibers and found that the mechanical properties decreased dramatically with increasing straw content compared to standard mortars by more than 30–40% of the corresponding values. Although these samples showed a significant decrease in the overall mechanical resistance, no actual collapse of the samples was observed after the plastic behavior described above, which was mainly found at higher straw contents. Pachla et al. [32] studied the sustainable application of rice straw in porous composites. They found that adding rice straw to porous composites reduced compressive strength by 15% while increasing three-point bending strength. Feng et al. [37] studied the effect of alkali-activated rice straw on composites. They found that alkali-activated straw fibers had a more significant impact on the composites than untreated rice straw. The amount of rice straw fiber added also had specific requirements, and an appropriate amount of straw fiber admixture could significantly improve the performance of modified cement samples. Still, excessive straw fibers would have adverse effects. Bentchikou et al. [38] studied the effect of recycled cellulose fibers on the performance of composite matrices and found that as fiber content increased, compressive strength decreased. This reduction was primarily due to the increase in voids caused by higher fiber content, which reduced the material’s weight and weakened it. However, the flexural strength did not exhibit a monotonic behavior with fiber content; it increased between 0% and 4% fiber content, possibly due to the bridging effect of the fibers within the matrix, but then gradually decreased. This latter behavior could be attributed to the combination of two potential phenomena: the effect of fibers on the uneven dispersion within the matrix and the weakening response due to the reduced volume proportion of the cement matrix. Soroushian et al. [39] reported that increasing the content of softwood fibers from 5% to 15% (fiber/cement matrix weight ratio) improved the system’s toughness. However, the flexural strength showed a slight increase (an average of 5%) when the fiber content increased from 5% to 10% and then decreased (an average reduction of 15%) as the fiber content increased to 15%. Sliva et al. [24] in their study on the performance of sisal fiber-cement composites found that when the length of sisal fibers increased from 10 mm to 40 mm, the strain failure rate decreased from approximately 5.2% to 2.6%. Simultaneously, the Weibull modulus decreased from 4.6 mm to 3.0 as the gauge length increased from 10 mm to 40 mm. Among the well-studied materials such as wood, sisal, hemp, coconut, bamboo, rice, and wheat straws, their common feature is their high cellulose fiber content. Cellulose fibers play an essential role in the performance of plant fiber-reinforced composites, offering excellent strength and durability. Additionally, cellulose fibers can increase the thermal insulation properties of composites, reducing heat conduction and improving insulation. They also have good moisture absorption and sound insulation properties.

This study found that alfalfa, one type of grass, also has a high cellulose fiber content. Grass is also an essential sustainable renewable biomass resource. China’s natural grassland area is nearly 6 billion m^2^, accounting for 41.7% of the country’s land area. Inner Mongolia’s grassland is the largest and most diverse ecological functional area in northern China, with an area of 88.44 million hectares, accounting for 22% of the national grassland area. Inner Mongolia’s planting area of silage corn, alfalfa, forage oats, and sheep grass is nearly 1.2 million hectares, yielding over 20 million tons. The natural grassland forage yield exceeds 30 million tons. However, most grass withers and degrades naturally on the grassland, with only a small amount used as animal feed. Therefore, accelerating the comprehensive utilization of grass resources in China has become an urgent task for Chinese researchers [40]. Grass grows year-round, but grass straw is rarely used as a building material. Applying grass to building materials will provide new avenues and directions for developing grass resources.

This study aims to determine the feasibility of using grass fibers to reinforce cementless slag-based composites through experiments. It also analyzes the effects of grass fibers and quicklime on the properties of the composites. It optimizes the mixture proportions to enhance the mechanical properties of alkali-activated slag-based composites.

## 2. Experiments and Methods

### 2.1. Materials and Sample Preparation

#### 2.1.1. Materials

The slag-based grass fiber blocks comprise grass straw (alfalfa straw), alkali-activated granulated blast furnace slag powder, quicklime, fine aggregates, water, and additives.

- Grass

**Figure 1:**
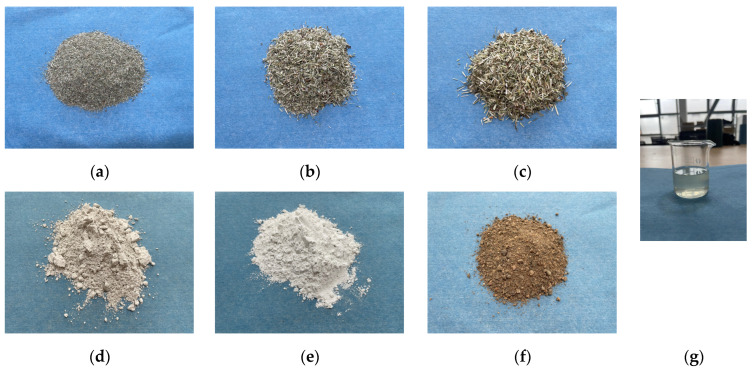
(**a**) 1 mm grass (**b**) 2 mm grass (**c**) 3 mm grass (**d**) Slag (**e**) Quicklime (**f**) Sand (**g**) Additives.

The grass selected is alfalfa, as shown in Figure 1, and it was collected from a pasture in Inner Mongolia. The grass is typically harvested when it is lush but not overly mature. The harvested grass is spread out and dried to dehydrate, requiring 3 days of sun drying. The grass must be turned regularly to ensure even drying and prevent mold. The dried grass is then crushed using a grinder into particles with lengths of 1–3 mm and classified using a standard sieve. As shown in Figure 1a–c In this experiment, the content and size of grass were two of the main factors studied; where the content of grass was taken as values of 5 g, 10 g, and 15 g; and the size of grass was taken as values of 1 mm, 2 mm and 3 mm.

- Slag

Granulated blast furnace slag powder made by Gongyi Longze Water Purification Materials Co., Ltd., Gongyi, China, is the slag that is utilized. It is rated S105 for strength. As shown in Figure 1d. Table 1 and Table 2 show the chemical composition and pertinent characteristics that meet the testing standards as specified in the Chinese national standard for ground granulated blast furnace slag used for cement and concrete (GB/T 18046-2017) [41]. In this test, the amount of slag was fixed at 300 g.

- Quicklime

The quicklime used in this experiment meets the requirements as specified in the Chinese national standard JCT 621-1996 [42] for lime used in silicate building products. The quicklime appearance is shown in Figure 1e. In this experiment, the amount of quicklime was also one of the variables studied, the amount of quicklime used was 27.5 g, 37.5 g, and 47.5 g.

- Fine aggregate

The fine aggregate used in the experiment is natural river sand, as shown in Figure 1f. with a bulk density typically at 1.48 × 10^3^ kg/m^3^ and a fineness modulus generally ranging between 2.5 and 3.5. The diameter of the fine sand particles ranged from 0.075 mm to 0.425 mm. It complies as defined by the Chinese national standard for construction sand (GB/T 14684-2011) [43] and according to the ASTM standard specification for concrete aggregates (ASTM C33/C33M-18) [44]. In the experiment, the amount of fine aggregate-fine river sand was 100 g.

- Additives

The additive used in this experiment is a concrete early strength agent. As shown in Figure 1g. Its main components include silicate, gypsum, and calcium oxide. These components can enhance the reactivity of the composite material and promote the hydration reaction. In the experiment, the amount of modified material additive was 18 g.

- Water

The water used in the experiment is regular tap water from the teaching building, with a pH of 6.5. In the experiment, the amount of water used was 225 g.

#### 2.1.2. Sample Preparation

**Figure 2:**
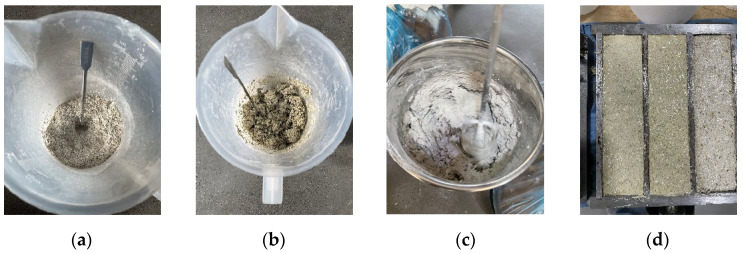
The process of making grass bricks. (**a**) Mix grass, slag, quicklime and sand dry ingredients well; (**b**) First time you add water; (**c**) Second addition of water; (**d**) Pour the mixture into the molds, level and compact it.

The preparation process for the grass fiber slag-based composite material is as follows: weigh the required amounts of alfalfa straw, slag, quicklime, sand, additive, and water for the specimen. (1) Weigh the grass fibers, slag, quicklime, and sand, and mix them evenly as shown in Figure 2a. (2) Add water in two stages: First, pour water into the mixture and continuously stir manually until all materials are thoroughly wetted, as shown in Figure 2b. Then, add the remaining water and start mixing with a mixer, as shown in Figure 2c, with a mixing time of 3 min. (3) After the mixture is uniform, add the additives and mix with the mixer for an additional minute. (4) Clean the sample molds. After cleaning and drying, apply a thin layer of mineral oil to the inner walls of the molds. Pour the mixture into the molds, which have dimensions of 40 × 40 × 160 mm. Level and compact the mixture with vibration as shown in Figure 2d. Prepare three samples for each group of tests. To prevent moisture evaporation, cover the samples with plastic film. (5) Allow the samples to set in the molds for two days. Then, de-mold the samples and place them in foam boxes in the laboratory for curing (at 20 °C ± 2 °C, 50% ± 5% RH) for 56 days. During the curing process, spray the blocks with water using a spray bottle every two days.

**Figure 3:**
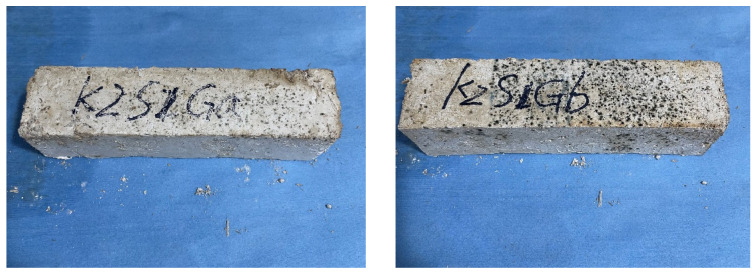
Picture of moldy grass bricks in the front.

In the initial round of experiments, the test groups consisted of slag, grass, sand, additives, and water. During the curing process, it was observed that the grass bricks exhibited varying degrees of mold growth, as shown in Figure 3. After reviewing the literature and analyzing the causes, it was found that grass contains cellulose, hemicellulose, lignin, pectin (Pectin is broken down into sugars and used as a nutrient by bacteria), wax layers, ash, proteins, and other components [45,46,47,48]. These organic substances serve as excellent nutrients for microorganisms, promoting their growth and reproduction. The warm and humid environments further facilitate the microbial decomposition of fats, waxes, and other easily degradable organic materials present on the untreated fiber surfaces. Moreover, Pectin and waxes in the cell walls of plant fibers also inhibit the interlocking of fibers within the matrix, resulting in poor adhesion between the fibers and the matrix. Consequently, this leads to reduced strength performance and inefficient stress distribution [49,50,51]. For the treatment of plant fibers, existing research has proposed various techniques, including treatments with benzoyl chloride, alkali, acetic anhydride, potassium permanganate, acetic acid, silane, and peroxides [52,53,54]. These treatment techniques aim to improve the mechanical properties of plant fibers by altering their crystallinity and removing weaker components from the fiber surface, such as fats, lignin, and pectin [55]. Therefore, in this experiment, quicklime was added to the original mix. Quicklime is a binding material for the blocks and affects the grass explicitly. When quicklime reacts with water, it forms calcium hydroxide, a strong alkali. The grass fibers eroded by the strong alkali lose moisture due to the dissolution of a large amount of sugar-like substances, causing them to shrink. The smooth wax layer on the surface of the fibers is corroded and roughened [56], which improves the physical properties of the plant fibers and enhances the bonding of substances inside the blocks.

### 2.2. Performance Test Experiments

#### 2.2.1. Microstructural Observations

- X-ray diffractometer

The dried grass powder was characterized using an X-ray diffractometer (model D8, Advance). Whenever the experimental material was sieved through a standard sieve with a mesh size of 70–80, 0.5 g of sample was added. The scanning range of the sample was set between 50° and 80°, with a scanning speed of 10°/min.

- Scanning Electron Microscope (SEM)

The samples’ porous structure and microstructure were visually analyzed using the HITACHI-S-3400N by Hitachi High-Technologies Corporation in Tokyo, Japan scanning electron microscope (SEM). Using image acquisition in low vacuum mode, the microscopic surface structure of the composite material particles was investigated. The experimental setup included an acceleration pressure range of 0.3–30 kV, a maximum sample diameter of 200 mm, and a low vacuum mode backscattered electron (BSE) resolution of 30 kV. Test samples with a diameter smaller than 10 mm were wrapped on five sides with tin foil, leaving only the top surface exposed for observation. Subsequently, they were fixed to glass slides using conductive adhesive. Before testing, the samples underwent gold sputtering using an ion-sputtering instrument. The samples that had been gold-plated were put on a holder and vacuum-treated. Appropriate magnification was selected to obtain SEM images of the respective materials.

#### 2.2.2. Physical Property Tests

- Apparent density

This study employed the procedure outlined in BS EN 1015-10 [57] to measure the sample density. The mass of the sample prism was obtained by weighing it on a micro-digital balance. Subsequently, the samples were uniformly dried at approximately 60 °C ± 5 °C until a continuous weight was reached within 2 h. The density was then determined by dividing the mass by the prism’s volume.

- Apparent porosity

The apparent porosity is calculated using Equation (4) [58]:

$P_{a}$ represents the apparent porosity (%), W_A_ is the water absorption (%), $\rho_{a}$ is the apparent density (kg/m^3^), and $\rho_{w}$ is the density of water (kg/m^3^).

(1) $$P_{a}=\frac{W_{A}\times \rho_{a}}{\rho_{w}\times 100}$$

- Water absorption

The water absorption (W_A_) coefficient, caused by the capillary action of the tested material, was measured at 20 °C [59]. The composite material samples were immersed in 5–10 mm of water for 24 h until fully saturated [60]. Two samples of each composite material were prepared for testing, and the average of the two results was taken.

W_A_ represents the water absorption (%), W_1_ is the weight of the dried sample (g), and W_2_ is the weight of the saturated sample (g).

(2) $$W_{A}=\left(\frac{W_{2}-W_{1}}{W_{1}}\right)\times 100$$

#### 2.2.3. Mechanical Property Tests

- Flexural strength

**Figure 4:**
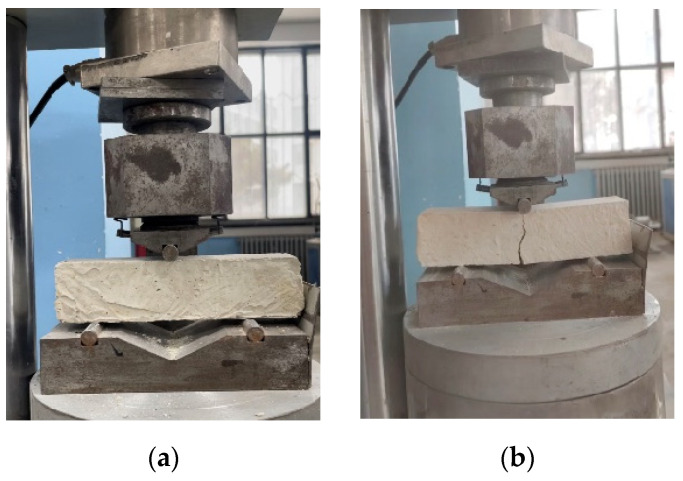
Flexural strength test Compressive strength. (**a**) Place the test block on the instrument; (**b**) Test block destroyed.

The flexural strength was measured using the central loading method described in the standard as per the Chinese national standard for test methods of mechanical properties of ordinary concrete (GB/T 50129-2011) [61] for testing the basic mechanical properties of masonry. An automatic cement flexural and compressive testing machine (DYH-300B, manufactured by Shandong Luda Testing Equipment Manufacturing Co., Ltd., Tai’an, China) was used to test the mechanical properties of the specimens; As shown in Figure 4a. Each group consisted of three samples with dimensions of 40 mm × 40 mm × 160 mm. After forming the specimens, they were placed in the testing machine with the surface facing up. The loading speed was set to 50 N/s. The specimen is subjected to a bending load until a visible crack appears, as shown in Figure 4b. The test is repeated three times for each specimen. The average of the three measurements was taken as the flexural strength of the sample.

**Figure 5:**
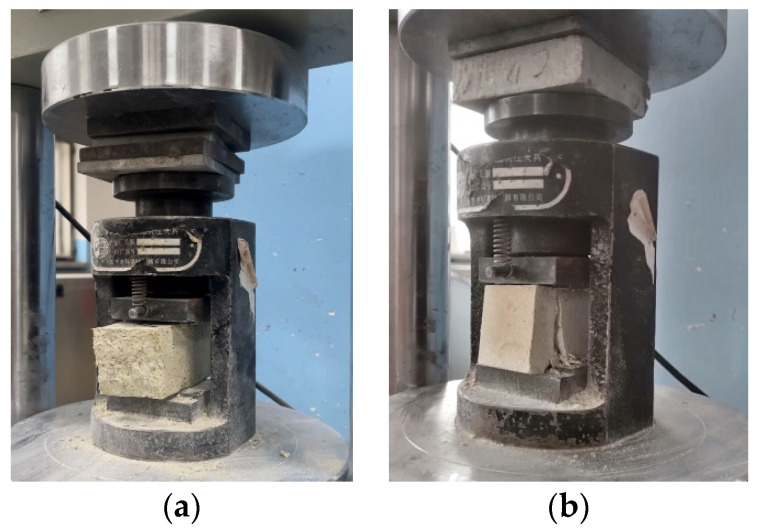
Compressive strength test. (**a**) Place the test block on the instrument; (**b**) Test block destroyed.

The compressive strength was determined using the central loading method described in the standard as per the Chinese national standard for test methods of mechanical properties of ordinary concrete (GB/T 50129-2011) [61] for testing the basic mechanical properties of masonry. An automatic cement flexural and compressive testing machine (DYH-300B, manufactured by Shandong Luda Testing Equipment Manufacturing Co., Ltd., Tai’an, China) was employed to test the mechanical properties of the specimens. The blocks obtained from the flexural tests (six per group) were used for the compressive strength tests. As shown in Figure 5a. Before placing the samples into the fixtures, the surface debris was removed. The sides of the specimens were used as the compression surfaces, and the loading speed was set to 1 kN/s. The specimen is subjected to a compressive load from the testing machine until failure occurs. As shown in Figure 5b. The average of the three test results was taken as the compressive strength.

### 2.3. Response Surface Method (RSM)

RSM is an optimization technique that measures a system’s reaction in relation to one or more variables. It employs graphical methods to illustrate functional correlations, enabling the selection of ideal circumstances for experimental design by intuitive observation [62,63]. RSM considers experimental random errors and fits complex, unknown functional relationships within a small region using a quadratic polynomial model to obtain continuous predictive models. These models allow for continuous evaluation at every stage of this study. Before using RSM, a single-factor experiment was conducted to study the effects of different proportions of three factors—grass content (5 g–15 g), grass size (1–3 mm), and quicklime content (27.5 g–47.5 g)—on the physical and mechanical properties of the composite material.

Using the Box-Behnken response surface optimization design, each factor in the Box-Behnken design method has three levels; each level is coded as (−1, 0, 1). The design matrix is centered at 0, with +1 and −1 representing the high and low values of the cube points, respectively. Through single-factor trials, appropriate ratios of grass content, grass size, and quicklime content were found for the experimental design. The statistical program Design Expert 10.0.3 was utilized in this investigation. Thirteen matching schemes and four repeat matching schemes were part of the experimental design, and the experimental variables were coded. The general form of the predictive polynomial equation is:

(3) $$Y=\beta_{0}+\sum_{i=1}^{K}\beta_{i}X_{i}+\sum_{i=1}^{K}\beta_{ii}\overset{2}{\underset{i}{X}}+\sum_{i=1}^{K-1}*\sum_{j=1}^{K}\beta_{ij}X_{i}X_{j}$$

where Y represents the response variables (flexural strength (MPa), compressive strength (MPa), water absorption rate (%) in this case; X_i_ and X_j_ are the independent variables (i, j = 1, 2, 3, …, k); and β_0_, β_i_, β_ii_, β_ij_ are the regression constant coefficients, linear coefficients, second-order coefficients, and interaction coefficients, respectively. The mathematical model was fitted using an analysis of variance (ANOVA); significance tests were run on linear, second-order, and third-order models to choose the best model. Using an ANOVA, the *p*-values for the model and each of its terms were determined in order to assess the statistical significance. Tests for significance and consistency were conducted to evaluate further the model’s validity, including residual vs. predicted value analysis, residual analysis, and predicted vs. observed value tests. An appropriate model that passes all necessary statistical tests can be used to predict responses or optimize processes [64]. Plotting response surface graphs and contour maps, which illustrate the correlations between factors and response values, was done using the generated data after it had been fitted to the model. The best process parameters for the material were found by modifying the additive optimization settings, and they were then experimentally confirmed.

## 3. Results and Discussion

### 3.1. Single-Factor Experimental

#### 3.1.1. Effect of Grass Size, Grass Content, and Quicklime Content on Compressive Strength

**Figure 6:**
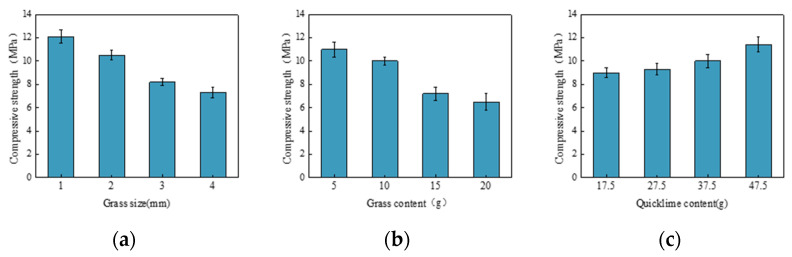
(**a**) Effect of grass size on compressive strength; (**b**) Effect of grass content on compressive strength; (**c**) Effect of quicklime content on compressive strength.

Figure 6 shows the compressive strength test results from the single-factor experiment. The changes in grass size, as in Figure 6a and grass content, as in Figure 6b, exhibit similar patterns. The compressive strength of the samples shows a clear trend: it gradually decreases with the increase in grass size or grass content. Adding grass increases the composite material’s porosity, and excessive porosity decreases compressive strength. Additionally, as the amount of grass increases, the slag matrix cannot effectively bond with the grass fibers, weakening its mechanical properties.

On the other hand, adding quicklime leads to a gradual increase in compressive strength, as in Figure 6c. This is because quicklime reacts with the water in the composite material, creating a cementing effect and forming a solid structure. The calcium hydroxide further reacts with carbonate ions in the water to form calcium carbonate crystals, enhancing the block’s strength and hardness. This process addresses the issue of low early compressive strength and further improves the strength of the composite material.

#### 3.1.2. Effect of Grass Size, Grass Content, and Quicklime Content on Flexural Strength

**Figure 7:**
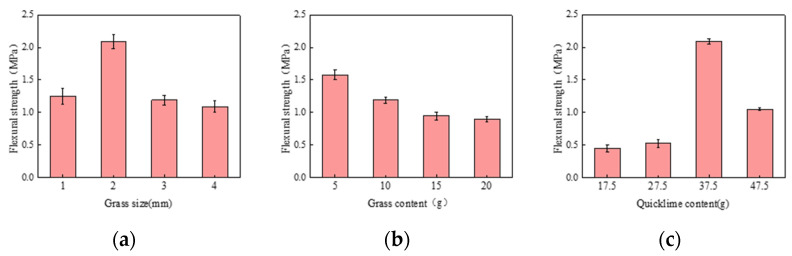
(**a**) Effect of grass size on flexural strength; (**b**) Effect of grass content on flexural strength; (**c**) Effect of quicklime content on flexural strength.

Figure 7 illustrates the flexural strength test results from the single-factor experiment. The variations in grass size, as in Figure 7a and quicklime content, as in Figure 7c, follow similar trends. The flexural strength of the samples first increases and then decreases with the increase in grass size or quicklime content. The samples achieve maximum flexural strength when the grass size is 2 mm. Beyond this threshold, the flexural strength decreases, likely because appropriately sized grass fibers enhance the material’s toughness, thus increasing flexural strength. However, excessively long fibers result in an uneven internal structure, increasing cracks and defects, which reduce strength.

Similarly, the flexural strength reaches its optimum when the quicklime content is 37.5 g. If the quicklime content is too low, it cannot adequately react with the base material. Conversely, an excessively high quicklime content makes the composite material overly dense, causing internal stress concentration and reducing flexural strength. Furthermore, as the grass content, as in Figure 7b, increases, the flexural strength gradually decreases.

#### 3.1.3. Effect of Grass Size, Grass Content, and Quicklime Content on Water Absorption Strength

**Figure 8:**
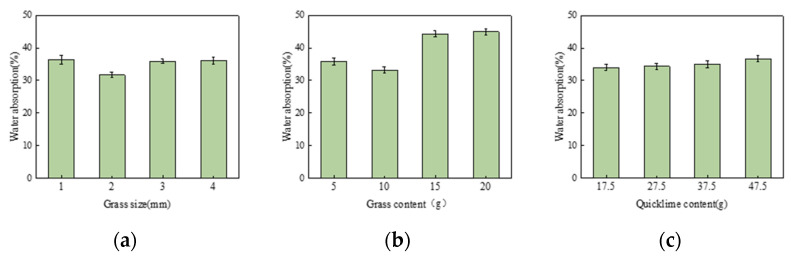
(**a**) Effect of grass size on water absorption rate; (**b**) Effect of grass content on water absorption rate; (**c**) Effect of quicklime content on water absorption rate.

Figure 8 shows the water absorption test results from the single-factor experiment. The variation in grass size, as in Figure 8a, exhibits a trend of initially decreasing and increasing water absorption. When the grass size is 2 mm, the water absorption rate is at its lowest, at 21.52%. This might be because smaller grass fibers react and bond more easily with the slag matrix, reducing water penetration. In contrast, larger grass fibers may have more gaps, increasing water penetration and, thus, water absorption. Both the grass content, as in Figure 8b, and the quicklime content, as in Figure 8c, show similar trends in their effect on water absorption: as their content increases, water absorption also increases. The high grass content leads to higher water absorption due to the high cellulose content, which has strong hydrophilic properties. Quicklime also has strong water absorption characteristics, so as its content increases, water absorption likewise increases.

Therefore, the optimal ranges are determined: grass size between 1–3 mm, grass content between 5–15 g, and quicklime content between 27.5–47.5 g.

### 3.2. RSM Experiment

#### 3.2.1. Design of Experiment (DoE)

Based on the results of the single-factor experiments, Design Expert 10.0.3 software was used to perform a Box-Behnken design optimization. The factors were grass content, grass size, and quicklime content, with compressive strength, flexural strength, and water absorption as responses. An L17 (3^3^) orthogonal array was obtained, containing 17 sample groups, including 13 unique schemes and four repetitions. The level values for every design factor are displayed in Table 3. The design approaches indicated in Table 4 were followed in preparing the samples, which were then put through mechanical and physical testing. Table 4 presents the results of this study.

#### 3.2.2. Model Building and Testing

The data were fitted to polynomial models using variance analysis (ANOVA). Model *p*-values and consistency tests were used to perform significance tests for linear, quadratic, and cubic models in order to determine which model was appropriate. The quadratic model was shown to be the most significant based on the results. The obtained second-order polynomial regression model is as follows:

Y_α_ = 10.6 − 1.87 × A − 1.42 × B + 1.77 × C + 0.22 × AB + 0.73 × AC + 0.27 × BC − 0.91 × A^2^ − 1.31 × B^2^ − 1.46 × C^2^ Y_β_ = 2.08 + 0.24 × A − 0.11 × B + 0.16 × C − 0.2 × AB − 0.028 × AC − 0.12 × BC − 0.46 × A^2^ − 0.68 × B^2^ − 0.71 × C^2^ Y_γ_ = 22.02 − 0.54 × A + 2.5 × B + 2.85 × C − 0.29 × AB + 0.72 × AC − 1.1 × BC + 2.89 × A^2^ + 1.77 × B^2^ + 4.4 × C^2^ (4)

where the grass size, grass content, and quicklime content are denoted as A, B, and C, respectively, and the responses of compressive strength (α), flexural strength (β), and water absorption (γ) are used for multiple regression fitting.

The optimum approach for confirming the relevance of the model is an analysis of variance (ANOVA), which was utilized to get the *p*-values for each model [65]. As shown in Table 5 through 7, the *p*-values of the regression models are all less than 0.0001, indicating that the regression models are statistically significant and highly significant. The *p*-values for the lack-of-fit terms (*p* = 0.2592 in Table 5, *p* = 0.1650 in Table 6, *p* = 0.3190 in Table 7) are all greater than 0.05, indicating that the lack-of-fit terms are not significant and the models have a good fit.

The *p*-values for the grass size, grass content, and quicklime content are all less than 0.05, indicating significant interactions among these three factors in the regression model. In Table 5, the primary terms of grass size, grass content, and quicklime content significantly impact compressive strength (*p* < 0.01). The main effects analysis shows the relationship among the factors: A > C > B, i.e., grass size > quicklime content > grass content. The second-order interaction term AC has a significant impact on compressive strength (*p* < 0.01), while BC and AB do not have significant effects (*p* > 0.05). The influence of the second-order interactions on compressive strength is ranked as AC > BC > AB.

In Table 6, the primary terms of grass size and quicklime content have a highly significant impact on flexural strength (*p* < 0.01), and grass size also significantly affects flexural strength (*p* < 0.05). The main effects analysis shows the relationship among the factors: A > C > B, i.e., grass size > quicklime content > grass content. The second-order interaction term AB has a significant impact on flexural strength (*p* < 0.05), while AC and BC do not have significant effects (*p* > 0.05). The influence of the second-order interactions on flexural strength is ranked as AB > BC > AC.

In Table 7, the primary factors of grass content and quicklime content have a highly significant effect on water absorption (*p* < 0.01), while grass size has a significant effect on water absorption (*p* < 0.05). The analysis of the main effects of each factor is ranked as follows: C > B > A, meaning quicklime content > grass content > grass size. The secondary interaction BC has a highly significant effect on water absorption (*p* < 0.01), AC has a significant effect on water absorption (*p* < 0.05), and AB has no significant effect on water absorption (*p* > 0.05).

**Figure 9:**
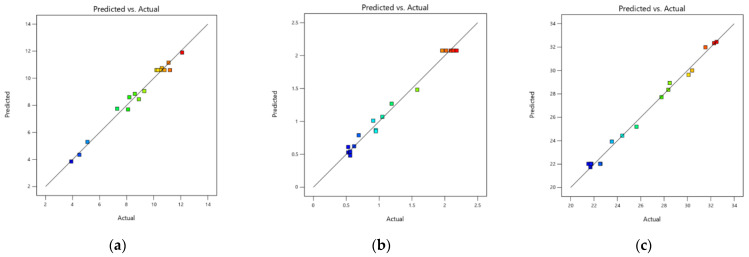
Diagnostic Plots: (**a**) Predicted versus actual graph of compressive strength; (**b**) Predicted versus actual graph of flexural strength; (**c**) Predicted versus actual graph of water absorption rate. (Blue indicates the lowest response, represents residuals that are small or negative. Green represents the median response, represents residuals that are close to zero, indicating good model fit. and red means the highest response peak in the plot, indicates large or positive residuals, showing significant deviation from the model’s predictions).

Additional methods for testing the model included the Pareto test, residual analysis, and comparison of anticipated and experimental values. Figure 9a–c display the predicted values versus the actual values for all models. These figures are used to check and evaluate all models’ correlation, goodness of fit, and predictive accuracy. A perfect fit model would have all data points aligned perfectly along the linear trend line in the prediction versus actual plots [66].

Figure 9a–c show that the data points for all models reasonably fit the drawn linear trend lines, indicating good predictive performance and model fit, with all transformed models having a correlation coefficient greater than 0.9. In Figure 9c, the data points for the slump model show the best fit, resulting in a line with a slope of 0.9826. In Figure 9a–c, the data points’ color explains each response’s ranking; blue indicates the lowest response, represent residuals that are small or negative., green represents the median response, represents residuals that are close to zero, indicating good model fit. and red means the highest response peak in the plot, indicates large or positive residuals, showing significant deviation from the model’s predictions [67,68].

#### 3.2.3. Multi-Factor Experimental Results

The related response surface and contour plots were created using the regression equations of the response values in order to further maximize the strength of the composite material [66]. The contour and three-dimensional response surfaces of the quadratic model’s polynomial regression equation represent the interaction results of various factors. These plots not only help predict and optimize the response values but also analyze the interaction effects between any two factors to understand their interaction patterns. Contour plots consist of multiple lines of equal response values, connecting neighboring points with the same response values into closed curves. The curvature of the response surface plots shows a positive correlation with the factors’ effect on the response values. The steeper the surface and the greater the curvature, the more significant the effect of the factor on the response value [69]. The curvature’s dimension is shown by the color change trend; the more the curvature, the faster the blue-to-red color shift. Response surface plots and contour plots are analyzed similarly. The intensity of the interaction effect between the two parameters is also determined by the contour lines’ form. When the contour pattern is circular, there is little interaction between the two elements and a similar influence on the response value. The two elements’ interaction is more significant if the form is elliptical [70].

**Figure 10:**
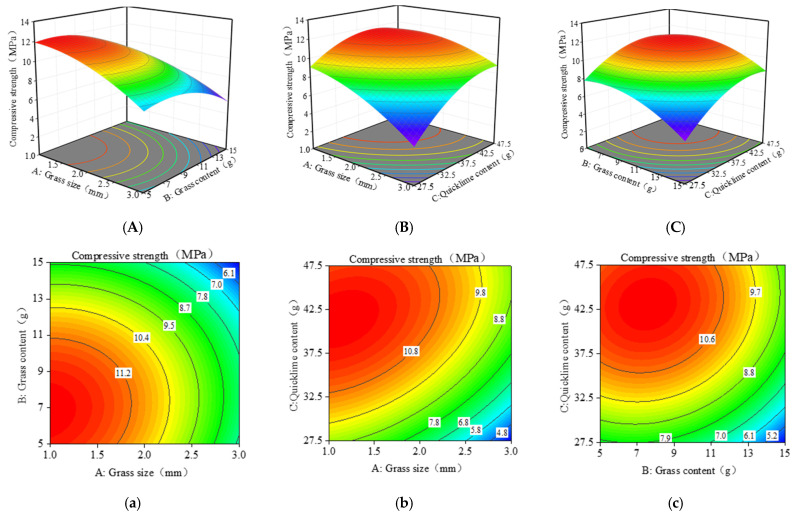
RSM model plots for compressive strength: (**A**–**C**) 3D response surface; (**a**–**c**) Contour line diagram of the model. (The blue to red color represents the change in output value from low to high. The numbers in the graph are generally used to label the response values at specific points or contours. The line in the figure represents the set of points on the response surface that have the same response value. The dots in the graph represent experimental or simulated data points).

Figure 10 illustrates the three-dimensional surface and contour plots showing the effects of three factors (grass size, grass content, and quicklime content) on the compressive strength of the composite material. The interaction between grass content and grass size on compressive strength is depicted in Figure 10a. The highest compressive strength is achieved when the grass size is between 1 and 1.5 mm, and the grass content is around 5–7 g. This observation is consistent with the analysis results in Table 5.

The interaction between grass content and quicklime content on compressive strength is shown in Figure 10B,b. The compressive strength increases with a decrease in grass content and an increase in quicklime content up to a certain threshold. Beyond this threshold, the compressive strength starts to decrease. The maximum compressive strength is achieved when the grass content is around 5–7 g, and the quicklime content is around 42.5–47.5 g. Figure 10C,c displays the impact of grass size and quicklime content on compressive strength. When the quicklime content is low, the compressive strength decreases with increased grass size. When the quicklime content is high, the compressive strength initially remains stable and then decreases with increased grass size. The highest compressive strength is observed when the grass size is between 1.0 and 1.5 mm and the quicklime content is around 42.5–47.5 g.

**Figure 11:**
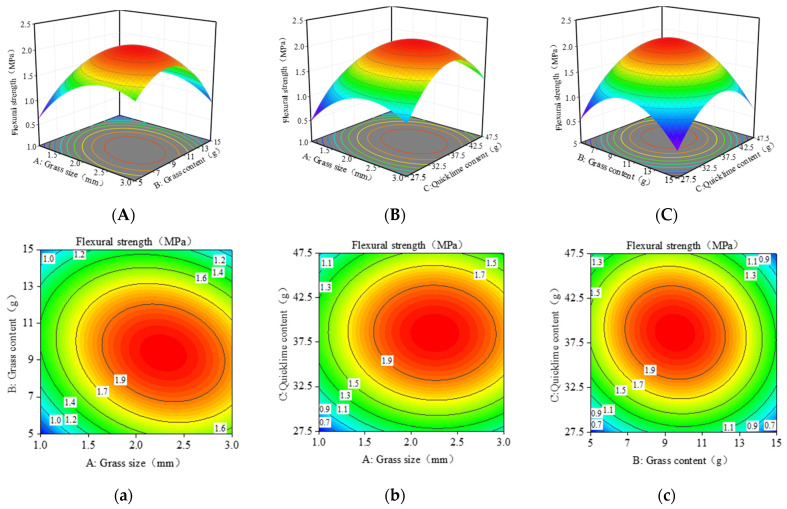
RSM model plots for flexural strength: (**A**–**C**) 3D response surface; (**a**–**c**) Contour line diagram of the model. (The blue to red color represents the change in output value from low to high. The numbers in the graph are generally used to label the response values at specific points or contours. The line in the figure represents the set of points on the response surface that have the same response value. The dots in the graph represent experimental or simulated data points.).

In Figure 11, the contour shapes indicate that the interaction between grass content and grass size is the most significant, which is consistent with the analysis results in Table 6.

In Figure 11A,a, the slope of grass size is steeper than grass content, indicating that grass size has a more significant impact on flexural strength. This is because the appropriate length of grass fibers can bond the bricks, thus affecting their flexural strength. The maximum flexural strength is achieved when the grass size is between 2–3 mm and the grass content is around 7–13 g.

Figure 11B,b shows the effect of quicklime content and grass content on flexural strength. In the three-dimensional surface plot, the slope change of quicklime content is steeper, indicating that quicklime has a more significant effect on flexural strength than grass content. The flexural strength gradually improves and then falls as the amount of grass and quicklime increases. When the quicklime content is between 32.5 and 42.5 g and the grass content is between 7 and 13 g, the maximum flexural strength is attained.

Figure 11C,c shows the effect of quicklime content and grass size on flexural strength. In the three-dimensional plot 11-C, the slopes of grass size and quicklime content are comparable, indicating that both factors significantly affect flexural strength. The impact of quicklime on flexural strength is mainly due to the stress within the bricks, which influences the flexural strength. When the values reach a certain threshold, the material becomes too dense, causing internal stress concentration, which reduces flexural strength. The maximum flexural strength is observed when the grass size is 1.5–2.5 mm and the quicklime content is around 32.5–42.5 g.

**Figure 12:**
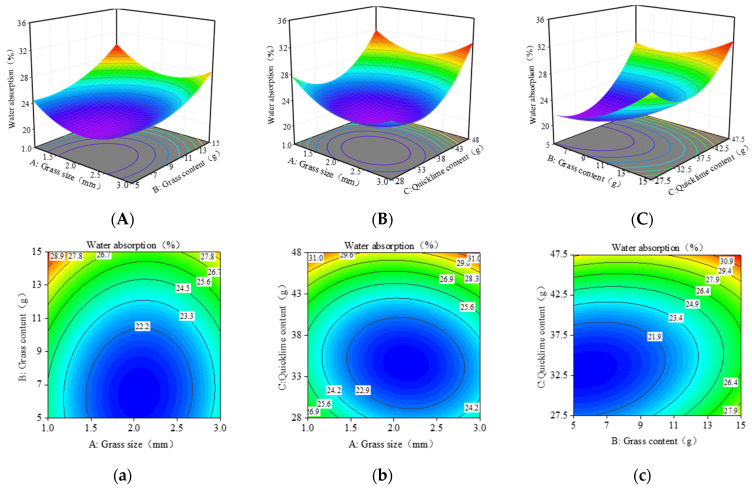
RSM model plots for water absorption: (**A**–**C**) 3D response surface; (**a**–**c**) Contour line diagram of the model. (The blue to red color represents the change in output value from low to high. The numbers in the graph are generally used to label the response values at specific points or contours. The line in the figure represents the set of points on the response surface that have the same response value. The dots in the graph represent experimental or simulated data points.).

In Figure 12, the contour shapes reveal that the interaction between grass content and grass size is the most significant, which aligns with the analysis results in Table 7.

In Figure 12A,a, the slope of grass content is steeper than grass size, indicating that grass content significantly impacts water absorption capacity. The minimum water absorption rate is achieved when the grass content is between 5 and 9 g and the grass size is around 1.5 and 2.5 mm.

Figure 12B,b shows the effect of quicklime content and grass content on water absorption capacity. In the three-dimensional surface plot, the steep slope associated with quicklime content indicates its significant influence on water absorption capacity, compared to grass content. Initially, the water absorption rate remains constant with increasing quicklime content, then rises. At low quicklime content, the interaction between quicklime and grass fibers is weak, leading to a gradual increase in water absorption as grass content rises. Conversely, at high quicklime content, the interaction strengthens, allowing grass fibers to enhance their water absorption capacity fully. The minimum water absorption rate is achieved when the quicklime content is between 27.5 and 42.5 g and the grass content is around 5–9 g.

Figure 12C,c shows the effect of quicklime content and grass size on water absorption capacity. In the three-dimensional plot 12-C, the slope of quicklime content is steeper than that of grass size, indicating that quicklime significantly impacts water absorption capacity. Quicklime inherently has strong moisture absorption properties, hence its strong water absorption capacity. When the quicklime content is low, the water absorption rate initially decreases and then slowly increases with an increase in grass size, with a relatively small increase. When the quicklime content is high, the water absorption rate initially decreases slightly and increases significantly with increased grass size. This is primarily because smaller grass fibers react and bind more easily with quicklime, reducing water permeability and lowering the water absorption rate. Larger grass fibers may have more gaps, increasing water permeability and thus increasing the water absorption rate. The minimum water absorption rate is achieved when the quicklime content is 32.5–42.5 g and the grass size is around 1.5–2.5 mm.

#### 3.2.4. Optimization and Validation

To maximize the workability and mechanical strength of the composite material while minimizing its water absorption rate, a multi-objective optimization method from Response Surface Methodology (RSM) was employed. The optimization aimed to achieve the desired parameter proportions and potential combinations by setting specific goals for each variable and response. Table 8 summarizes the optimization criteria and objectives for these variables and responses, focusing on maximizing compressive strength and flexural strength while minimizing water absorption. During the optimization, the grass size, grass content, and quicklime content were maintained within 1–3 mm, 5–15 g, and 27.5–47.5 g, respectively.

As shown in Table 8, setting the grass size to 2 mm with a grass content of 8.4 g and a quicklime content of 38 g is the best choice for producing composite materials.

To confirm the accuracy of the optimization results, composite material blocks were produced utilizing the ideal process parameters of quicklime content, grass size, and grass content. The compressive strength, flexural strength, and water absorption rate were measured at 11.425 MPa, 2.145 MPa, and 21.89%, respectively. The relative errors between these experimental values and the predicted values of total compressive strength (11.019 MPa), flexural strength (2.042 MPa), and water absorption rate (21.538%) were within an 8% range. This confirms the excellent correlation between predicted and experimental values, indicating that the optimal processing technique obtained through the response surface methodology is reasonable.

### 3.3. Analysis of Microscopic Performance

#### 3.3.1. X-ray Diffraction

**Figure 13:**
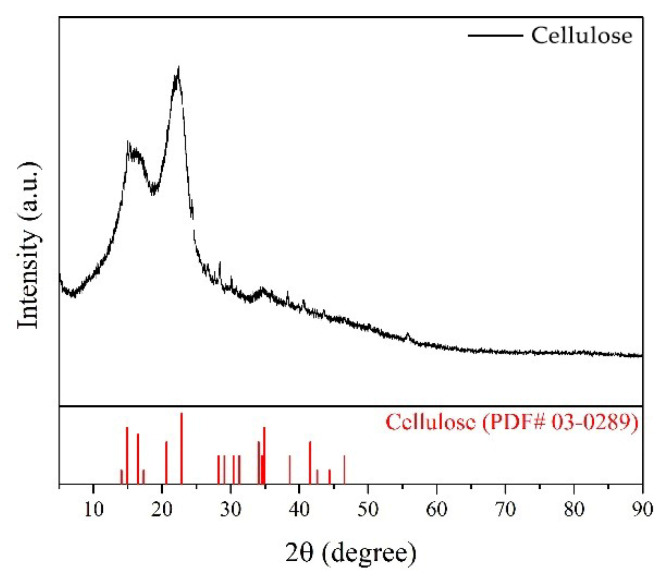
XRD pattern of alfalfa grass.

X-ray analysis was conducted on the grass fibers to investigate the crystalline structure of cellulose in the grass, aiming to observe the overall impact of grass on the mechanical properties of the composite material. As shown in Figure 13, the XRD pattern of the sample exhibits a series of characteristic diffraction peaks that can be attributed to natural cellulose (PDF# 03-0289). Precisely, the diffraction peaks near 2θ = 16.5°, 22.8°, and 34.9° correspond to the (-111), (002), and (040) crystal planes of natural cellulose, respectively. These results indicate that the sample is primarily composed of cellulose. Organic cellulose molecules are polar, with strong intermolecular forces and the ability to form hydrogen bonds between molecules. This leads to the crystallization of multiple cellulose chains into insoluble microfibrils and the formation of two structural regions: crystalline and amorphous. X-ray diffraction (XRD) can quantitatively measure the crystalline regions in cellulose. By analyzing the intensity and position of the diffraction peaks, we can calculate the crystallinity index, which is the ratio of the crystalline region to the amorphous region. The higher the crystallinity of the cellulose, the stronger and more durable the material. For grass fiber-reinforced slag-based composites, higher crystallinity results in greater structural integrity and load-bearing capacity. All characteristics impart high strength, stiffness, durability, and biocompatibility to cellulose [71]. In particular, intramolecular hydrogen bonds prevent the rotation of glycosidic bonds, significantly increasing rigidity. Using fibers with a high cellulose content to produce building materials can positively affect their mechanical properties [72]. Furthermore, because cellulose is biodegradable, using it in the construction industry may reduce the negative environmental effects of construction waste by acting as an effective means for the recycling and utilization of resources.

#### 3.3.2. Micromorphology

**Figure 14:**
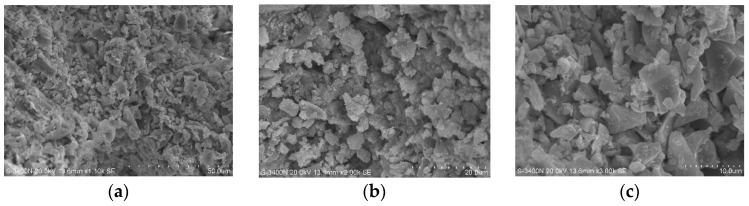

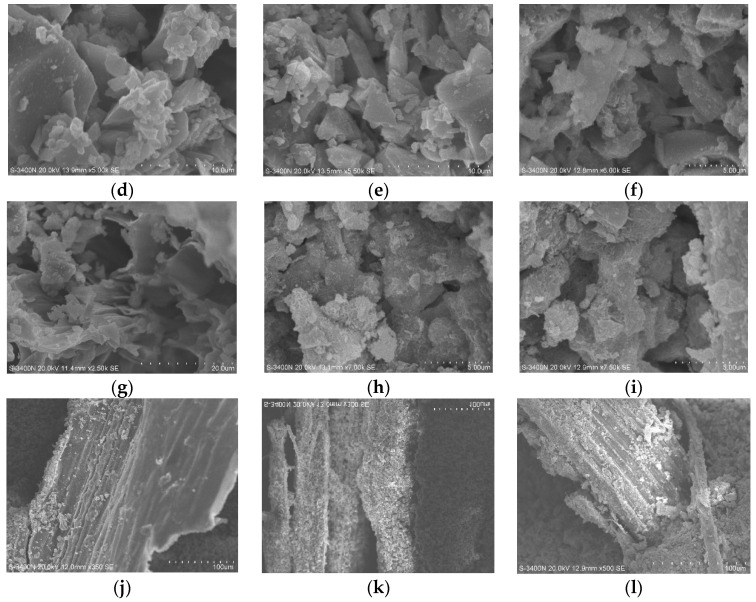
SEM images of grass fiber slag-based composites. ((**a**–**i**) show the relationship between the various materials in the composite at the microscopic level); (**j**–**l**) show the morphology of the grasses at the microscopic level in the composite).

The hydration of quicklime produces calcium hydroxide, which chemically reacts with the surface of grass fibers, causing changes to their surface. Additionally, the hydration reaction generates a significant amount of heat, which may also affect the internal structure of the grass fibers. SEM experiments were conducted on the composite materials to observe the synergistic effects of the microscopic structures of the various materials. SEM is typically used to observe the fine structure of materials that ordinary microscopes cannot resolve, examining the microscopic morphology of samples to analyze their influence on mechanical properties. The changes in the components after the hydration reaction and the microscopic images of the pores and grass fibers in the composite materials were observed under a scanning electron microscope (SEM). The mechanical strength of the plant fiber blocks is primarily determined by the interaction between the Ca(OH)_2_ matrix, formed after the hydration reaction of quicklime with grass straw, and the slag matrix. As shown in Figure 14a–i, the Ca(OH)_2_ and slag powder raw materials appear as loosely packed, porous structures with relatively large pore sizes.

The grass straw fibers serve as the dispersed phase, while Ca(OH)_2_ and slag powder form the continuous phase. After being molded into bricks, the Ca(OH)_2_ and slag are compressed, resulting in a relatively dense structure. Moreover, this process reduces the gaps between fibers, increasing the contact area. The straw fibers appear to be embedded within the dense Ca(OH)_2_ powder in the bricks [73]. It is suggested that this may be due to the formation of primary metal complexes through hydrogen bonding between Ca^2+^ ions and the OH^−^ functional groups in cellulose and lignin, as indicated in previous studies. The Ca(OH)_2_ particles may concentrate on cellulose’s inner and outer surfaces, as proposed by [73,74]. However, this interpretation is based on earlier research and has not been directly verified in the current study. From Figure 14j–l, it can be observed that the surface of the grass fibers is relatively rough, leading to a tighter integration with the slag matrix material, which positively influences mechanical properties.

Additionally, observations reveal that grass fibers are not easily discernible in the microscopic morphology of the composite material, likely due to the better homogeneity of the fiber material within the composite, resulting in a reduced pore volume [60].

## 4. Conclusions

In this study, the response surface methodology (RSM) was used to examine the effects of three key factors—grass size, grass content, and quicklime content—on three response variables: compressive strength, flexural strength, and water absorption rate. This approach also helps determine the optimal levels of these factors and facilitates predictive modeling and optimization of the mixture ratios. The conclusions are as follows:

1. The effects of grass size, grass content, and quicklime content on compressive strength, flexural strength, and water absorption rate were studied through single-factor experiments. Based on these results, the influencing factors and levels for the response surface experiments were determined: the range of grass size is 1–3 mm, the range of grass content is 5–15 g, and the range of quicklime content is 27.5–47.5 g.
2. A second-order polynomial regression equation was used to fit the experimental data, establishing a response surface model to describe the relationship between the three factors and the three response variables. RSM predicts that when the grass size, grass content, and quicklime content are 2.0 mm, 8.2 g, and 38 g, respectively, the compressive strength and flexural strength reach their maximum values. In contrast, the water absorption rate reaches its minimum, precisely 12.1 MPa, 2.18 MPa, and 21.52%. The optimized experimental values were 11.425 MPa, 2.145 MPa, and 21.89%, respectively. The experimental and predicted values differ by only 8%, indicating a good fit.
3. The use of microstructural properties provides valuable insights into the characterization of materials. XRD analysis indicated that the primary component of alfalfa fiber is cellulose, which is hydrophilic and increases the water absorption rate of the composite material. This leads to the degradation of cellulose and results in poor stress transfer efficiency, subsequently decreasing the mechanical properties of the composite material. SEM experiments were conducted on well-performing specimens to analyze the internal structural characteristics of the composite material. The results showed that the grass material was not easily detected under microscopic observation, likely due to the better uniformity of the fiber material within the composite, which reduces pore volume. Additionally, the rough surface of the grass fibers facilitates better bonding with the matrix material, enhancing the density and strength of the composite.

## Figures and Tables

**Table 1:** Chemical composition of S105 slag.

Form	CaO	SiO_2_	Al_2_O_3_	MgO	Fe_2_O_3_	SO_3_
S105 slag	35.30	34.50	16.70	5.01	1.50	1.24

**Table 2:** Properties of S105 slag.

Specific Surface Area (m^2^/kg)	Flowability Ratio (%)	Activity Index 7d (%)	Activity Index 28d (%)	Density (g/cm^3^)	Loss on Ignition (%)	Water Content (%)
628.00	102.00	98.00	115.00	2.93	0.96	0.20

**Table 3:** Table of experimental design for 3-factor, 3-level response surface analysis.

Factor	Level		
	−1	0	1
A—Grass size (mm)	1	2	3
B—Grass content (g)	5	10	15
C—Quicklime content (g)	27.5	37.5	47.5

**Table 4:** Response surface optimization experimental design and results.

Test No.	A—Grass Size (mm)	B—Grass Content (g)	C—Quicklime Content (g)	Y1—Compressive Strength (MPa)	Y2—Flexural Strength (%)	Y3—Water Absorption Rate (%)
1	1	5	37.5	12.1	0.53	24.42
2	3	5	37.5	8.1	1.58	23.53
3	1	15	37.5	8.2	0.69	30.4
4	3	15	37.5	5.1	0.95	28.36
5	1	10	27.5	9.3	0.56	27.78
6	3	10	27.5	3.9	0.91	25.64
7	1	10	47.5	11.1	0.95	31.54
8	3	10	47.5	8.6	1.19	32.28
9	2	5	27.5	7.3	0.53	21.69
10	2	15	27.5	4.5	0.56	28.48
11	2	5	47.5	10.6	1.05	30.09
12	2	15	47.5	8.9	0.62	32.49
13	2	10	37.5	10.5	2.09	21.52
14	2	10	37.5	10.2	2.02	21.78
15	2	10	37.5	10.3	1.96	22.56
16	2	10	37.5	11.2	2.13	21.74
17	2	10	37.5	10.8	2.18	22.52

**Table 5:** Results of regression analysis for compressive strength model and regression coefficients.

Sources	The Sum of Squared Deviations	Degrees of Freedom	Mean Square	F-Values	*p*-Values Prob > F	Significant
Models	94.16	9	10.46	44.66	<0.0001	**
A—Grass size	28.12	1	28.12	120.05	<0.0001	**
B—Grass content	16.24	1	16.24	69.34	<0.0001	**
C—Quicklime content	25.2	1	25.2	107.58	<0.0001	**
AB	0.2	1	0.2	0.86	0.3835	
AC	2.1	1	2.1	8.97	0.0201	*
BC	0.3	1	0.3	1.29	0.2932	
A^2^	3.51	1	3.51	14.96	0.0061	**
B^2^	7.25	1	7.25	30.96	0.0008	**
C^2^	9.01	1	9.01	38.44	0.0004	**
Residual	1.64	7	0.23			
Lost proposal	0.98	3	0.33	1.98	0.2592	ns
Pure error	0.66	4	0.16			
Aggregate	95.8	16				

Note: *p* < 0.01 is highly significant and is denoted by **, *p* < 0.05 is significant and is denoted by *, *p* > 0.05 is not significant and is denoted by ns. R^2^ = 0.9829, Adj R^2^ = 0.9609.

**Table 6:** Results of regression analysis of flexural strength model and regression coefficients.

Sources	The Sum of Squared Deviations	Degrees of Freedom	Mean Square	F-Values	*p*-Values Prob > F	Significant
Models	6.46	9	0.72	51.82	<0.0001	**
A—Grass size	0.45	1	0.45	32.58	0.0007	**
B—Grass content	0.095	1	0.095	6.83	0.0347	*
C—Quicklime content	0.2	1	0.2	14.1	0.0071	**
AB	0.16	1	0.16	11.27	0.0121	*
AC	3.03 × 10^−3^	1	3.03 × 10^−3^	0.22	0.6544	
BC	0.053	1	0.053	3.82	0.0916	
A^2^	0.9	1	0.9	65.17	<0.0001	**
B^2^	1.92	1	1.92	138.73	<0.0001	*
C^2^	2.13	1	2.13	153.47	<0.0001	**
Residual	0.097	7	0.014			
Lost proposal	0.066	3	0.022	2.9	0.1650	ns
Pure error	0.031	4	7.63 × 10^−3^			
Aggregate	6.56	16				

Note: *p* < 0.01 is highly significant and is denoted by **, *p* < 0.05 is significant and is denoted by *, *p* > 0.05 is not significant and is denoted by ns. R^2^ = 0.9852, Adj R^2^ = 0.9662.

**Table 7:** Results of regression analysis for water absorption model and regression coefficients.

Sources	The Sum of Squared Deviations	Degrees of Freedom	Mean Square	F-Values	*p*-Values Prob > F	Significant
Models	267.08	9	29.68	101.17	<0.0001	**
A—Grass size	2.34	1	2.34	7.99	0.0255	*
B—Grass content	50	1	50	170.47	<0.0001	**
C—Quicklime content	65.04	1	65.04	221.73	<0.0001	**
AB	0.33	1	0.33	1.13	0.3236	
AC	2.07	1	2.07	7.07	0.0325	*
BC	4.82	1	4.82	16.43	0.0049	**
A^2^	35.12	1	35.12	119.73	<0.0001	**
B^2^	13.12	1	13.12	44.74	0.0003	**
C^2^	81.44	1	81.44	277.66	<0.0001	**
Residual	2.05	7	0.29			
Lost proposal	1.13	3	0.38	1.62	0.319	ns
Pure error	0.93	4	0.23			
Aggregate	269.13	16				

Note: *p* < 0.01 is highly significant and is denoted by **, *p* < 0.05 is significant and is denoted by *, *p* > 0.05 is not significant and is denoted by ns. R^2^ = 0.9924, Adj R^2^ = 0.9826.

**Table 8:** Optimization objectives for composite fabrication process variables.

Names	Units	Goals	Target	Level of Importance
A: grass size	mm	In range (1~3)	2	
B: grass content	g	In range (5~15)	8.4	
C: quicklime content	g	In range (27.5~47.5)	38	
Compressive strength	MPa	Maximize	11.019	1
Flexural strength	MPa	Maximize	2.042	1
Water absorption	%	Minimize	21.538	1

## Data Availability

No new data were created or analyzed in this study.
